# Successful Postoperative Nephrocutaneous Fistula Treatment With Omental Flap Grafting: A Case Report

**DOI:** 10.3389/fsurg.2021.728839

**Published:** 2021-11-11

**Authors:** Haonan Guan, Di Zhang, Xian Ma, Yechen Lu, Yiwen Niu, Yingkai Liu, Jiaoyun Dong, Yi Gao, Weiping Yang, Qimin Chen, Jiajun Tang, Shuliang Lu

**Affiliations:** ^1^Department of Burn, Ruijin Hospital, Shanghai Jiaotong University School of Medicine, Shanghai, China; ^2^Wound Healing Center, Ruijin Hospital, Shanghai Jiaotong University School of Medicine, Shanghai, China; ^3^Department of Urology, Ruijin Hospital, Shanghai Jiaotong University School of Medicine, Shanghai, China; ^4^Department of General Surgery, Ruijin Hospital, Shanghai Jiaotong University School of Medicine, Shanghai, China; ^5^Department of General Surgery, Shanghai Children's Medical Center, Shanghai Jiaotong University School of Medicine, Shanghai, China

**Keywords:** foreign body reaction, sinus tract, nephrectomy, omentum, nephrocutaneous fistula

## Abstract

Nephrocutaneous fistula (NCF) is a rare and severe complication of renal disease and surgical procedures. Treatments for NCF are based on the renal function, and can include nephrectomy, heminephrectomy, nephroureterectomy, endourological maneuvers or antibiotic therapy alone. Here we report a case of a chronic NCF which occurred 5 years after partial nephrectomy. In this report, we describe a new surgical approach for the management of a patient with postoperative NCF. In the present case, in addition to removing the fistulous tract, we also performed an omental flap grafting to tightly cover the kidney. In addition to limiting and controlling the local inflammation, the omental flap prevents contact between the kidney and the flank muscle on its posterior rim. No recurrence or complications occurred throughout 10 months of follow-up. The NCF was successfully treated with completely removal of the sinus tract and omental flap grafting, without nephrectomy. This case adds new aspects to the treatment of NCF.

## Introduction

Renal fistula to the adjacent organs is a common phenomenon, however nephrocutaneous fistula (NCF) are rare. NCF refers to fistula that develop between the kidney and the skin surface. The main predisposing causes of NCF cases reported in the literature are surgical procedures, renal stones, trauma, xanthogranulomatous pyelonephritis and renal tuberculosis (1–6). Treatments for NCF are based on the renal function, and can include nephrectomy, heminephrectomy, nephroureterectomy, endourological maneuvers or antibiotic therapy alone (7, 8).

Here we report a case of chronic NCF which occurred 5 years after partial nephrectomy, and was successfully treated with omental flap grafting, without re-nephrectomy.

## Case Presentation

**Figure 1:**
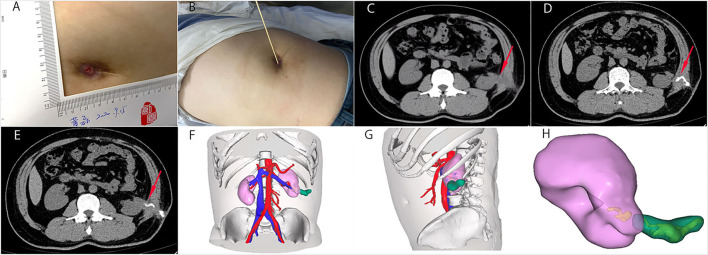
The general view and CT images of the nephrocutaneous fistula (NCF). **(A,B)** The fistula was 0.5 x 0.5 cm size and 6 cm deep on his left flank. **(C)** Abdominal CT showed the sinus tract (red arrow), extending from the surface of left kidney near the lower upper pole to the skin without renal/ureteral stone or hydronephrosis. **(D,E)** We injected contrast media into the sinus tract from the skin opening under pressure. The direction of the sinus tract is shown by the red arrow. **(F–H)** The three-dimensional reconstruction showed the relationship between the sinus tract and the left kidney. The sinus tract was marked green. We can see that the bottom of the sinus tract was closely connected with the lower pole of the left kidney.

In September 2020, a 39 year-old man was admitted to Shanghai Ruijin Hospital complaining of a serious discharge from a small opening on his left flank. It had been occurring for 2 months without fever or pain. His medical history evidenced a laparoscopic left partial nephrectomy for clear cell renal cell carcinoma (ccRCC) in December 2015. Three weeks post-surgery, he had developed purulent discharge from the drainage tube orifice on his left flank. Seven months later, after three rounds of debridement and percutaneous drainage procedures, the wound was healed. There was no report of previous tuberculosis or pyelonephritis. Physical examination revealed that the fistula covered an area of 0.5 x 0.5 cm, and 6 cm deep on his left flank without tenderness or percussed pain (Figures 1A,B). Laboratory tests including renal function tests and urinalysis showed no unusual results. Urine culture and secretion culture were negative. To rule out tuberculosis, we conducted a fluorescent quantitative PCR test of the secretions from the fistula, which was also unremarkable.

**Figure 2:**
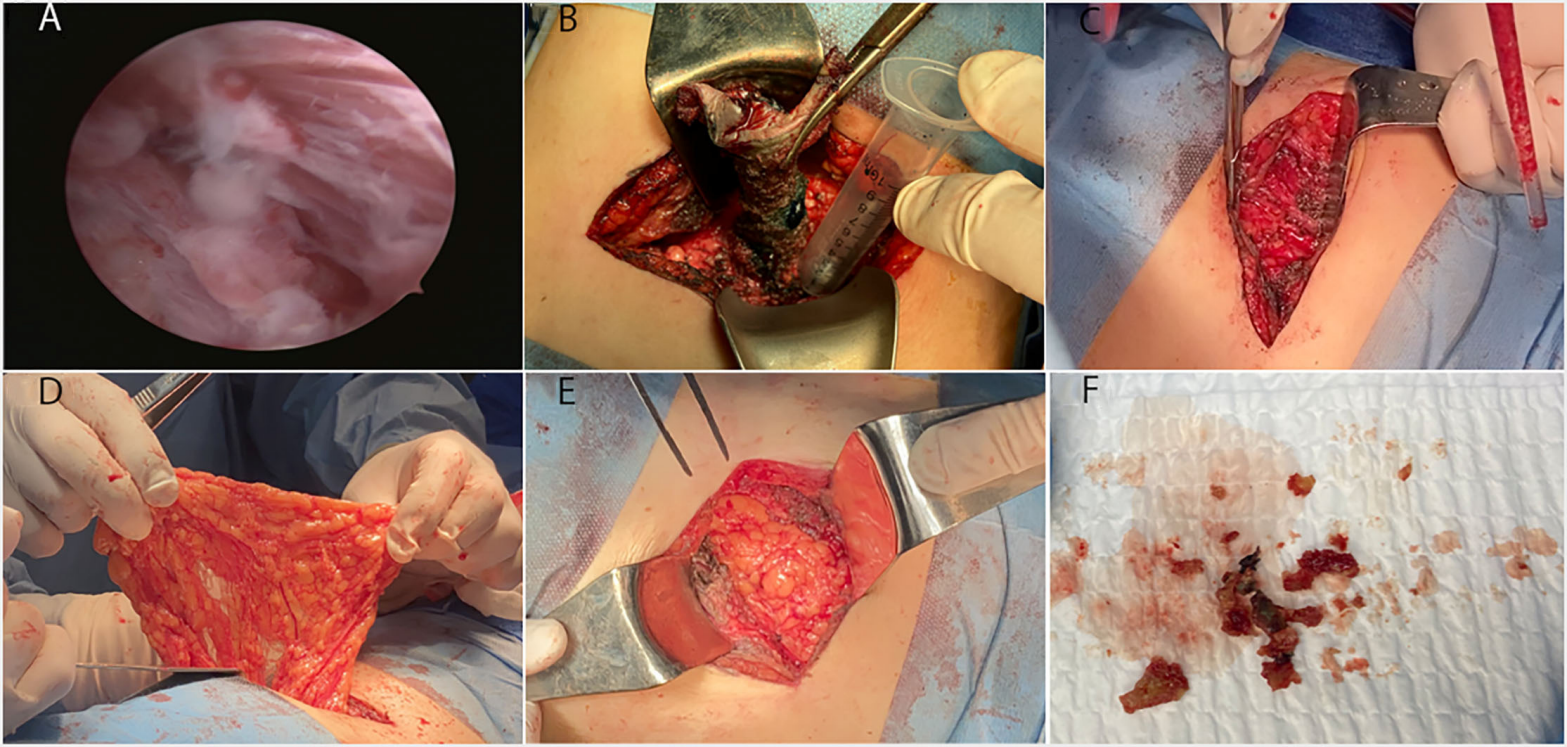
The fistulous tract endoscopic images and manifestation of surgery**. (A)** The fistulous tract endoscopy showed a mass of white flocculus stuck to the lining of the sinus tract. Because of the twist of the tract, our hard endoscopy cannot reach the bottom of the sinus tract. **(B)** Complete excision of the sinus tract under the guidance of methylene blue staining. The length of the tract was 6 to 7 cm. **(C,F)** A substantial amount of hard tissue similar to calcification and a hemoclip at the lower pole of the left kidney were founded. Removed all of these foreign objects. **(D)** Opened the posterior peritoneum and pulled part of the greater omentum tissue into the retroperitoneal space with oval forceps. **(E)** Tightly covered the left kidney with the omental flap.

Renal dynamic imaging revealed normal function of the left kidney. Preoperative abdominal CT showed the sinus tract, extending from the surface of left kidney near the lower upper pole to the skin without renal/ureteral stone or hydronephrosis (Figures 1C–E). The three-dimensional reconstruction clearly showed the relationship between the sinus tract and the kidney (Figures 1F–H). Next, we performed a fistulous tract endoscopy, which showed a mass of white flocculus stuck to the lining of the sinus tract (Figure 2A). Given the frequent recurrence and complexity of NCF, we planned to repair the fistula and prevent relapse using complete sinus tract resection combined with omental flap grafting.

Specific steps in the surgical operation were as follows:

1. Complete excision of the sinus tract under the guidance of methylene blue staining. The bottom of the sinus tract was stuck to the lower pole of the left kidney (Figure 2B).
2. We found a substantial amount of hard tissue similar to calcification, and a hemoclip at the lower pole of the left kidney. We removed all the abnormal tissue and hemostasis (Figures 2C,F).
3. We opened the posterior peritoneum and pulled part of the greater omentum tissue into the retroperitoneal space with oval forceps. Then, we tightly covered the left kidney with the omental flap and fixed it with sutures (Figures 2D,E).
4. We sutured the posterior peritoneum with the root of the omental flap. Then, we closed the posterior peritoneum incision. Finally, we closed the operation incision in layers in the usual manner.

**Figure 3:**
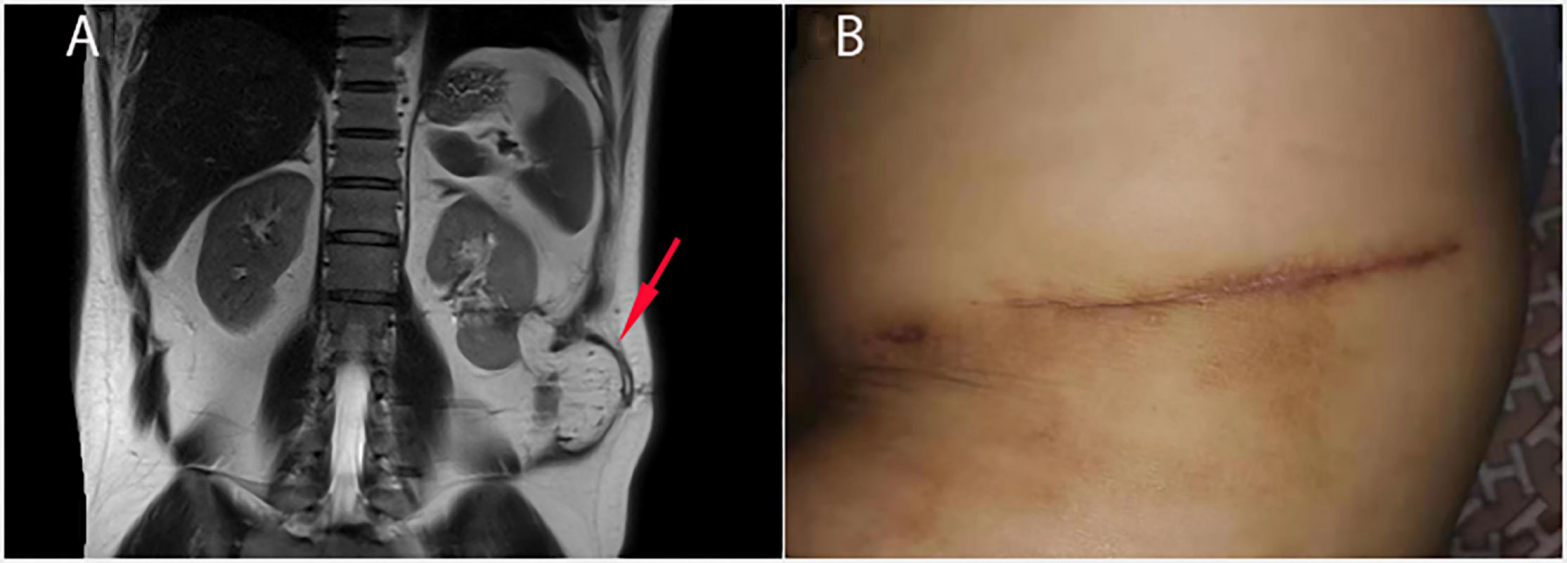
Follow-up 6 months after the operation. **(A)** MR examination showed that the omental flap tightly covered the left kidney (red arrow) and cut the kidney off from the flank muscle. **(B)** The NCF and the surgical incision were completely healed.

The pathological investigation of the resected sinus tract showed chronic inflammation with granulation hyperblastosis and foam cells growth. The patient was discharged 6 days after the operation. Six months later, MR examination showed that the NCF had healed well with the omental flap tightly covering the kidney (Figures 3A,B). The patient was asymptomatic throughout 12 months of follow-up.

## Discussion

NCF is a rare and severe complication of renal disease and surgical procedures. The kidney is a retroperitoneal organ surrounded by the Gerota's fascia and perinephric fat, which are the only intervening layers between the kidney and the flank muscle (9). However, through the lumbar region with anatomical weakness, such as the Petit's triangle, a fistulous tract originating from the kidney can easily reach the flank skin (1). The main predisposing causes of NCF include calculous disease, surgical procedures, renal tuberculosis, chronic pyelonephritis and renal trauma. Among them, surgical procedure has been reported as the most common pathogenesis of NCF, especially in developed countries (6, 8–11). In the present case, the patient had undergone laparoscopic left partial nephrectomy 5 years before, and most of the perinephric fat and capsule had been removed during this operation. Three weeks after the surgery, NCF appeared with purulent discharge. It was cured after 7 months of antibiotic treatment and surgical drainage. However, the NCF recurred 5 years later.

Surgical therapy is a crucial treatment for NCF. This is because the renal parenchyma is mostly involved accompanied by severe inflammation in most cases of diagnosed NCF. Therefore, it is difficult to close the fistula and prevent relapse without surgical treatments (1, 8). Surgical approaches which have been include nephron-sparing (NS) surgery and non-nephron-sparing (NNS) surgery (1, 8, 12). NNS surgery, like nephrectomy and nephroureterectomy, are the most common procedures, because in 90% of NCF patients, the kidneys are non or poorly functioning (1). NNS surgery includes partial nephrectomy, heminephrectomy, and simple fistulous tract resection for functioning kidneys (2, 4–6, 13, 14). In addition, endourological maneuvers such as positioning a double-J stent, percutaneous fulguration of the fistulous tract and nephrostomy have also been reported in 2 cases (15, 16). In both cases, the kidneys were functioning properly and little of the renal parenchyma was involved. However, most patients with NCF have undergone several surgeries, and repeated surgical stimulation can lead to chronic inflammation of local tissues, as well as the urinoma, which often leads to NCF recurrence.

The great omentum is a large visceral adipose tissue attached to the great curve of the stomach and the proximal part of the duodenum. Previous studies had revealed that the great omentum contains copious lymphocytes, macrophages, mast cells, stromal cells and growth factors (17–20). Also, studies have shown that the omentum facilitates liver regeneration and slows progression of CKD (21, 21, 22). Because of these properties, the omentum functions like an organ, and its biological functions include regulating local immune responses, controlling inflammation and infection, and promoting tissue repairment and regeneration (21, 21–24). Omental flap grafting has been used to repair conditions such as chronic ulcers (25, 26), head and neck defects (27, 28), hemifacial atrophy (29). Specially, omentum flap grafting has also been used for the treatment of various fistulas such as bronchopleural fistula (30), rectovesical fistula (31) and vesicovaginal fistula (32). However, to date, no cases of NCF treated with omental flap grafting have been reported.

In the present case, preoperative testing suggested that the collecting system was not involved. Hence ureteral stent placement and percutaneous drainage were unnecessary neither before nor after the procedure. Given that the involved kidney was still functioning, we decided to perform the NNS procedure. Interestingly, during our operation, we found a substantial amount of hard tissue similar to calcification, and a hemoclip, at the lower pole of the left kidney. Combined with the patient's previous surgical records and allergic constitution, we suspect that the hard tissue was the medical glue used in a previous operation. Moreover, the pathophysiology included a foreign body reaction around the medical glue and the hemoclip, which may have resulted in the NCF pathogenesis. Similarly, previous studies have reported that foreign body reaction caused by medical glue eventually leads to non-healing wounds, phlebitis, cellulitis and other complications (33–35). Also, we found that most of the perinephric fat and capsule were removed, and the residual perinephric tissue had severe inflammation with fibrosis. In this case, because of Long-term (5 years) foreign body reaction, the local immue environment had been changed to be pro-inflammatory and immuno-unbalanced. In addition, there was insufficient perinephric tissue for covering and protect renal repair. Based on our experience, had only fistulous tract resection and sutures been performed, there would have been a high risk of subsequent leakage and recurrence due to existing inflammation. Hence, in addition to removing the fistulous tract, we also performed an omental flap grafting to covered the kidney. We did this to control the local inflammation, absorb the drainage and repair the damaged renal parenchyma. Also, the omental flap can stick to the kidney, preventing urine leakage and contact between the kidney and the flank muscle. To date, the patient has remained asymptomatic throughout 12 month of follow-up, demonstrating the efficacy and safety of our treatment.

In conclusion, NCF still represents a severe and rare renal disease, and the omental flap grafting was a novel and effective surgical treatment which prevented nephrectomy and recurrence. As such, NCF caused by foreign body reaction due to medical glue is worthy of attention and further study.

## Data Availability Statement

The raw data supporting the conclusions of this article will be made available by the authors, without undue reservation.

## Ethics Statement

Written informed consent was obtained from the individual(s) for the publication of any potentially identifiable images or data included in this article.

## Author Contributions

HG, SL, and JT: concept and design. HG and DZ: drafting of the manuscript. WY, QC, and YG: surgical technical support. HG, DZ, and YLi: statistical analysis. JD and YN: administrative and material support. SL: supervision. All authors: critical revision of the manuscript for important intellectual content and acquisition, analysis, or interpretation of data.

## Conflict of Interest

The authors declare that the research was conducted in the absence of any commercial or financial relationships that could be construed as a potential conflict of interest.

## Publisher's Note

All claims expressed in this article are solely those of the authors and do not necessarily represent those of their affiliated organizations, or those of the publisher, the editors and the reviewers. Any product that may be evaluated in this article, or claim that may be made by its manufacturer, is not guaranteed or endorsed by the publisher.
